# Capturing the systemic immune signature of a norovirus infection: an n-of-1 case study within a clinical trial

**DOI:** 10.12688/wellcomeopenres.11300.2

**Published:** 2017-07-18

**Authors:** Antony J. Cutler, Joao Oliveira, Ricardo C. Ferreira, Ben Challis, Neil M. Walker, Sarah Caddy, Jia Lu, Helen E. Stevens, Deborah J. Smyth, Marcin L. Pekalski, Jane Kennet, Kara M.D. Hunter, Ian Goodfellow, Linda S. Wicker, John A. Todd, Frank Waldron-Lynch

**Affiliations:** 1 JDRF/Wellcome Trust Diabetes and Inflammation Laboratory, Wellcome Trust Center for Human Genetics, Nuffield Department of Medicine, National Institute for Health Research Oxford Biomedical Research Centre, University of Oxford, Oxford, OX3 7BN, UK; 2 JDRF/Wellcome Trust Diabetes and Inflammation Laboratory, Department of Medical Genetics, National Institute for Health Research Cambridge Biomedical Research Centre, Cambridge Institute for Medical Research, University of Cambridge, Cambridge Biomedical Campus,Cambridge, CB2 0XY, UK; 3 Wellcome Trust/MRC Institute of Metabolic Science, Department of Medicine, National Institute for Health Research Cambridge Biomedical Research Centre, University of Cambridge, Cambridge, CB2 0QQ, UK; 4 Division of Virology, Department of Pathology, University of Cambridge, Addenbrooke’s Hospital, Cambridge, CB2 0QQ, UK; 5 National Institute for Health Research Cambridge Clinical Trials Unit, Cambridge University Hospitals NHS foundation Trust, Cambridge Biomedical Campus, University of Cambridge, Cambridge, CB2 0QQ, UK; 6 Experimental Medicine and Immunotherapeutics, Department of Medicine, National Institute for Health Research Cambridge Biomedical Research Centre, University of Cambridge, Addenbrooke’s Hospital, Cambridge, CB2 0QQ, UK

**Keywords:** clinical trial, immune response, norovirus, T regulatory cells, aldesleukin, Proleukin, Interleukin-2

## Abstract

Background: The infection of a participant with norovirus during the adaptive study of interleukin-2 dose on regulatory T cells in type 1 diabetes (DILT1D) allowed a detailed insight into the cellular and cytokine immune responses to this prevalent gastrointestinal pathogen.

Methods: **Serial blood, serum and peripheral blood mononuclear cell (PBMC) samples were collected pre-, and post-development of the infection. To differentiate between the immune response to norovirus and to control for the administration of a single dose of aldesleukin (recombinant interleukin-2, rIL-2) alone, samples from five non-infected participants administered similar doses were analysed in parallel.

Results: Norovirus infection was self-limited and resolved within 24 hours, with the subsequent development of anti-norovirus antibodies. Serum pro- and anti-inflammatory cytokine levels, including IL-10, peaked during the symptomatic period of infection, coincident with increased frequencies of monocytes and neutrophils. At the same time, the frequency of regulatory CD4 ^+^ T cell (Treg), effector T cell (Teff) CD4 ^+^ and CD8 ^+^ subsets were dynamically reduced, rebounding to baseline levels or above at the next sampling point 24 hours later.  NK cells and NKT cells transiently increased CD69 expression and classical monocytes expressed increased levels of CD40, HLA-DR and SIGLEC-1, biomarkers of an interferon response. We also observed activation and mobilisation of Teffs, where increased frequencies of CD69 ^+^ and Ki-67 ^+^ effector memory Teffs were followed by the emergence of memory CD8 ^+^ Teff expressing the mucosal tissue homing markers CD103 and β7 integrin. Treg responses were coincident with the innate cell, Teff and cytokine response. Key Treg molecules FOXP3, CTLA-4, and CD25 were upregulated following infection, alongside an increase in frequency of Tregs with the capacity to home to tissues.

Conclusions: **The results illustrate the innate, adaptive and counter-regulatory immune responses to norovirus infection. Low-dose IL-2 administration induces many of the Treg responses observed during infection.

## Introduction

We conducted an interleukin-2 (IL-2; aldesleukin [Proleukin]) dose-finding, mechanistic trial, “Adaptive study of IL-2 dose on regulatory T cells in type 1 diabetes” (DILT1D), in 40 participants with type 1 diabetes (T1D), as a first step towards treatment and prevention of T1D by enhancing Treg functions ^1, 2^. Adverse events were recorded throughout the trial and one participant developed an acute gastrointestinal infection, confirmed as norovirus, in the period of daily sampling post-IL-2 injection. Noroviruses cause approximately 1 in 5 of all diarrhoea cases ^3^ and are a leading cause of acute viral gastroenteritis worldwide. In immunocompetent individuals, norovirus infection is contained within the gastrointestinal tract ^4^ and induces little pathology to the mucosal tissues ^5^, although complications such as necrotising enterocolitis, post-infectious irritable bowel syndrome or exacerbation of inflammatory bowel disease may occur. The immune response to norovirus infection is only partly understood, largely due until recently to the lack of suitable animal models and difficulty in growing human norovirus in cell culture. Nevertheless, it is becoming clear that autophagy, innate and adaptive immune responses are involved in the control of murine norovirus infection ^6^. A functional immune system is imperative for acute control of norovirus infection in humans ^7^, and pre-existing anti-norovirus IgA antibody titers negatively correlate with severity of symptoms in human challenge studies ^8^. Mouse models and human studies also suggest a significant role for type-I, type-II and type-III interferon responses in control of norovirus infection ^9, 10^.

The longitudinal design of the mechanistic trial, including frequent blood sampling and deep phenotyping protocols before and after IL-2 treatment, allowed thorough investigation of cellular responses and immune phenotypes associated with norovirus infection. Sampling the blood did not allow direct investigation of the mucosal immune response to norovirus infection. Nevertheless, the systemic nature of the response provided insight into the coincident effector cell activation and counter-regulatory immune response induced by norovirus infection.

## Methods

### Study design

The adaptive study of IL-2 dose on Tregs in T1D ( DILT1D) was a 60-day duration, single center, single dose non-randomised, open label, adaptive dose-finding trial that was conducted at the National Institute for Health Research Cambridge Biomedical Centre, Addenbrooke’s Hospital, Cambridge, United Kingdom ^1^. Potential participants were eligible for the study if they had a disease duration of less than 2 years, were positive for at least one autoantibody (anti-islet cell, anti-IA2, anti-ZnT8 and anti-GAD) and were between 18 – 50 years of age. The study had 12 visits: after a screening visit, the IL-2 dose (aldesleukin, [Proleukin], Novartis Pharmaceuticals Ltd UK) was subcutaneously administered on day 0, and the participants were then followed up and blood was taken at 90 minutes and days 1, 2, 3, 4, 7, 9, 14, 21 and 60 after administration of drug. Participants were clinically assessed before dosing and monitored for adverse events (AE) including infections at each subsequent time point. Samples of blood taken at each visit were subject to polychromatic flow cytometric immunophenotyping, serum samples were measured for biomarkers of immune activation and peripheral blood mononuclear cell (PBMC) samples were isolated and cryopreserved. The trial enrolled forty participants, and they were divided into five dose groups. In the 0.385-0.610 × 10 ^6^ IU/m ^2^ group, a single participant (IL-2 dose: 0.433 × 10 ^6^ IU/m ^2^) incidentally developed a norovirus infection 30 hours into the trial. The pretreatment and longitudinally collected samples from this participant and five other participants that received similar doses (dose range 0.408 – 0.445 × 10 ^6^ IU/m ^2^), were analysed to characterise the immune response to the pathogen and compare it to low dose IL-2 treatment alone. The study was sponsored by the University of Cambridge and Cambridge University Hospitals NHS Foundation Trust. Ethical approval was granted by the Health Research Authority, National Research Ethics Service, England (approval number: 13/EE/0020) and was registered in the ISRCTN Registry ( ISRCTN27852285) and at ClinicalTrials.gov ( NCT01827735). The detailed description of the protocol, design and rationale of DILT1D was published prior to analysis of this data ^2^.

### Cytokine and soluble receptor measurement

Serum or plasma cytokines were measured using electrochemiluminescence assays (Meso Scale Discovery [MSD], Rockville, Maryland, USA). IL-6 (plasma diluted 1:10) and IL-2 (plasma diluted 1:3) were measured at MSD, in quadruplicate, by the high-sensitivity S-PLEX assay. The IL-2 assay (limit of quantitation 2 fg/ml) fg/ml values were converted to IU/ml as detailed ^1^. Serum IL-12p70, IL-10 and TNF-α levels were measured using a multiplex assay using the V-PLEX proinflammatory panel 1 (human) kit (MSD). Samples were diluted 1:3 in proprietary buffer (MSD) and measured in duplicate. Plasma IFN-γ, IP-10 and CRP levels were measured in a single-plex assay using V-PLEX human IFN-γ, IP-10 (CXCL10) and CRP V-PLEX human kits, respectively (MSD). Samples were diluted 1:2, 1:4 or 1:1000, respectively and measured in duplicate. Data were acquired using a SECTOR S 6000 plate reader (MSD). 

Circulating soluble SIGLEC-1 (sSIGLEC-1) concentrations were measured in duplicate in plasma samples (diluted 1:10) using a non-isotopic time-resolved fluorescence ELISA assay based on the dissociation-enhanced lanthanide fluorescent immunoassay technology (DELFIA; PerkinElmer). Test plasma samples were measured on 96-well MaxiSorp microtiter plates (Nunc), and coated with 1 μg/ml monoclonal anti-human SIGLEC-1 antibody (clone HSn7D2; ABCAM). Detection was performed using a biotinylated sheep polyclonal anti-SIGLEC-1 (R&D Systems) diluted to a final concentration of 200 ng/ml in PBS + 10% FBS and a Europium-Streptavidin detection solution (PerkinElmer), diluted in standard DELFIA buffer. Quantification of test samples was obtained by fitting the readings to a human recombinant human SIGLEC-1 (R&D Systems) serial dilution standard curve plated in quadruplicate on each plate.

### Norovirus, vesivirus and hepatitis E virus antibody detection

Anti-norovirus, -vesivirus and -hepatitis E virus (HEV) antibody levels were measured by ELISA. Test serum samples were diluted 1:100 in PBS + 0.05% Tween 20 and were measured in duplicate on 96-well MaxiSorp microtiter plates (Nunc). They were coated overnight at 4°C with either 25 ng of norovirus (GII.4 Dijon) virus-like particle (VLP) (a kind gift from Alexis de Rougemont, Dijon, France), vesivirus (2117) VLP or HEV VLP (both generated in the Goodfellow laboratory) in 0.05 M carbonate/bicarbonate buffer (pH 9.6). Anti-viral antibodies were detected using horseradish peroxidase (HRP)-conjugated anti-human IgG antibody (Sigma Aldrich) diluted 1:10,000 in 5% milk PBS–Tween 20. Bound antibody was detected with tetramethylbenidine (TMB, Sigma Aldrich), the reaction was stopped with 1N H _2_SO _4_ and the optical density (OD) was read at 450 nm (Spectromax M2 plate reader, Molecular Devices).

### Norovirus RNA detection

RNA purified from 0.5 × 10 ^6^ PBMCs resuspended in RLT buffer (RNeasy micro kit, Qiagen, Germany) from each timepoint was tested using real-time qRT-PCR following the same protocol as in Kageyama *et al*. ^11^ using the GII primers/probes, as stated.

### Whole blood counts and flow cytometric analysis

Whole blood counts were carried out at the Pathology Partnership, Addenbrooke’s Hospital, Cambridge.

Clinical FACS:

The FACS assays used to measure T, B and NK cell counts and total Treg frequency was carried out according to good laboratory practice at the Clinical Immunology Laboratory, Department of Immunology, Addenbrooke’s Hospital, Cambridge, as described ^1^. 

***Whole blood surface staining***. Venous blood was collected in lithium heparin coated tubes (BD Biosciences). 100 μl of blood was stained with antibodies and brilliant violet staining buffer (Biolegend, San Diego, USA) for 40 minutes at room temperature. Stained blood samples were vortexed and the red blood cells lysed (FACS lysing solution, BD). The stained cells were resuspended in PBS (Life technologies, Paisley, UK), supplemented with 0.2 % BSA (Sigma-Aldrich, Gillingham, UK). See Supplementary Table 1 (panels 1–4) to see the panels used for surface staining.

***Intracellular/Intranuclear staining***. 150 μl of surface-stained blood was fixed and cells permeabilised using intranuclear staining Fixation/Permeabilization Buffer (Affymetrix eBioscience, Hatfield, UK). Cells were then stained in permeabilisation buffer (eBiosciences) as previously published ^12^ (see panel 5 in Supplementary Table 1).

***Cryopreserved PBMC staining***. PBMC aliquots were thawed at 37 ^°^C, transferred to a new tube and reconstituted with ice cold RPMI-1640 (Life Technologies) supplemented with 10% FBS in a dropwise fashion. PBMCs were washed twice in RPMI-1640 + 10% FBS. PBMCs were stained at room temperature for 40 minutes (see panels 7–9 in Supplementary Table 1). Cells were then incubated with Fixable Viability Dye eFluor780, (eBioscience) and fixed (FACS lysing solution, BD) prior to data acquisition.

***Phosphorylation of STAT5a staining***. Whole blood pSTAT5a staining was carried out as previously described ^13^ (see panel 6 in Supplementary Table 1).

All data were acquired on a Fortessa flow cytometer (BD) using FacsDiva software v6.2 (BD) and analysed using FlowJo software v9.6 (FLOWJO, LLC, Oregon, USA). 

## Results

### Clinical course

The index case in our study, presented with T1D aged 18, eight months prior to participation in DILT1D. At screening there was evidence of beta-cell autoimmunity with elevated titers of anti-GAD65 (92 U/ml [NR:1-5]) and anti-IA-2 (16 U/ml [NR:<7.5]), with preservation of some endogenous insulin production (random C-peptide 392 pmol/l [NR:174-960]), normal biochemical and hematological indices and no evidence of blood borne viruses. As detailed ^1^, prior to administration of IL-2, clinical history, examination and laboratory investigations were largely normal. The participant received a single dose of IL-2 subcutaneously (0.433 × 10 ^6^ IU/m ^2^) and had a normal clinical assessment 24 hours post-drug administration. Six hours later the participant first developed mild abdominal pain and nausea that persisted without metabolic deterioration (capillary blood glucose – 6.6 mmol). Forty-eight hours after administration, the participant, reviewed at home by the clinical trial team, reported ongoing abdominal discomfort and nausea but with good oral intake and adequate glucose control. That evening he deteriorated with progressive nausea, vomiting, sweats and limited oral intake accompanied by elevation in capillary blood glucose (> 25 mmol/l) with a concomitant rise in ketone levels. On the advice of the study team, he consumed 100 ml sugar-free fluid every 30 minutes to ensure adequate hydration and self-administered 10% of his total daily insulin requirement as a single subcutaneous dose of quick acting insulin to circumvent the onset of ketoacidosis. Within two hours of these interventions, capillary blood ketones were undetectable (< 0.3 mmol/l) and the participant was tolerating oral fluids, and a clinical diagnosis of norovirus infection was made. Twelve hours later, 72 hours following drug administration, symptoms and hyperglycemia were completely resolved.

### Induction of anti-norovirus antibody response

**Figure 1.**
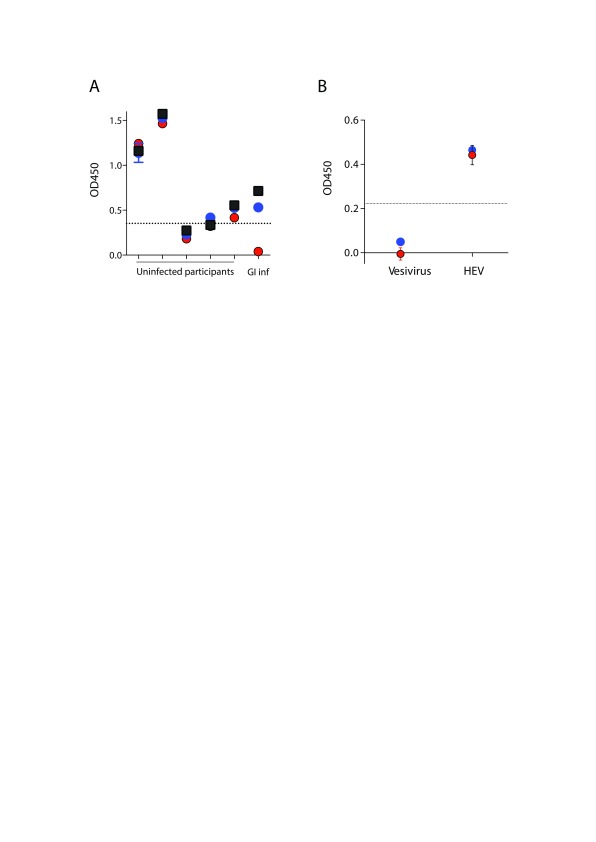
Specific increase in anti-norovirus GII.4 antibodies in the trial participant with gastrointestinal symptoms. ( **A**) Anti-norovirus GII.4 Dijon virus-like particles (VLP) serum antibody titres at day 0 (red filled circles), day 14 (filled black squares) and day 60 (filled blue circles) post-IL-2 dosing in six participants (5 dose-matched uninfected participants) and a participant with gastrointestinal symptoms receiving 0.408 – 0.445 × 10 ^6^ IU IL-2/m ^2^. ( **B**) Anti-vesivirus and hepatitis E virus (HEV) titres were assessed pre-IL-2 (filled red circle +/- SD) and day 60 post-IL-2 administration (filled blue circle +/- SD) in the infected participant.

To confirm the diagnosis, analysis of the serum for anti-norovirus antibodies was performed, showing an induction of anti-GII.4 IgG antibody titers post-infection ( Figure 1A) ^1^. We noted the low/undetectable level of pre-existing anti-norovirus antibodies, reflecting the blood group secretor status of the participant ^14^. The trial participant was homozygous for the 428G>A null mutation (rs601338 genotype AA) in *FUT2* and therefore does not express a functional α(1,2) fucosyltransferase enzyme that renders individuals largely, but not always, resistant to infection with GII.4 noroviruses ^15^. IL-2 treatment did not alter anti-GII.4 IgG titers in uninfected trial participants (n = 5) who had received a similar dose (range = 0.408 – 0.445 × 10 ^6^ IU IL-2/m ^2^) ( Figure 1A). Molecular testing for the GII genogroup norovirus RNA in PBMCs was negative at all visits in this participant, consistent with studies of immunocompetent adults in whom it is rare to detect norovirus RNA during infection ^4, 5^ ( Figures S1A and S1B). The trial protocol did not allow sampling of vomitus or fecal samples, precluding the direct demonstration of norovirus RNA in the participant. To exclude the possibility that the anti-GII.4 titers represented a broad non-specific anti-viral antibody response we tested serum against hepatitis E virus and vesivirus antigen and observed that serum IgG levels to both antigens were unchanged throughout the trial in the norovirus-infected participant ( Figure 1B).

### Cytokine and inflammatory marker responses

**Figure 2.**
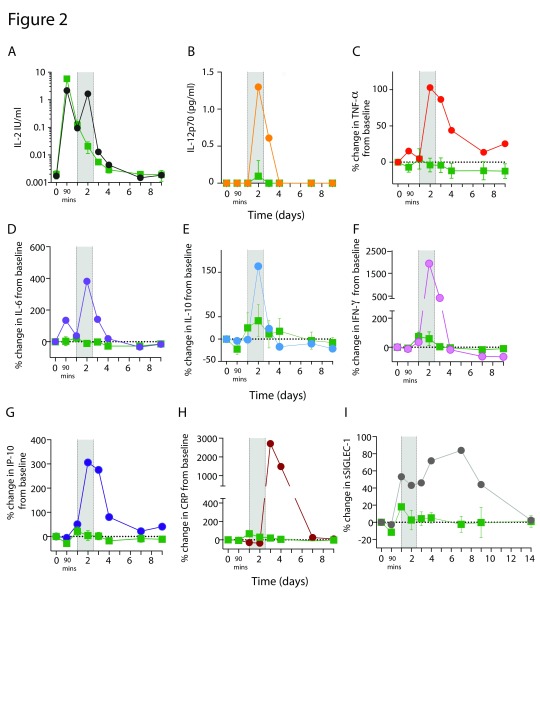
Norovirus infection induces proinflammatory cytokine and sSIGLEC-1 release. ( **A**) IL-2 levels (IU/ml) (black circles), ( **B**) IL-12p70 levels (pg/ml) (orange circles), ( **C**) the percentage change in TNF-α levels from baseline (red circles), ( **D**) IL-6 (violet circles), ( **E**) IL-10 (blue circles), ( **F**) IFN-γ (pink circles), ( **G**) IP-10 (purple circles), ( **H**) CRP (brown circles) and ( **I**) soluble SIGLEC-1 levels (grey circles) were measured in the norovirus-infected participant versus uninfected participants (green squares +/- SEM). Analytes were measured in serum or plasma. ( **B**– **H**) Data were normalised to and expressed as percentage change from baseline (day 0). The shaded area indicates the period of reported gastroenteritis.

The intensive longitudinal sampling in the DILT1D protocol allowed for measurement of serum cytokines/inflammatory markers pre- and post-norovirus infection within the affected participant. The inflammatory responses to the pathogen in the affected participant could also be compared to five control participants from the same dose group, enabling a comparison between antiviral and IL-2 drug responses. A primary increase in IL-2 levels (2.17–6.74 IU/ml) was observed in all participants at the 90 minute sampling point post drug administration, concordant with the systemic distribution of the drug ( Figure 2A), whereas a secondary peak of IL-2 (1.64 IU/ml) at day 2 was only detected in the infected participant. Infection induced an early increase in IL-12p70 levels (0–1.3 pg/ml) ( Figure 2B) and increases above baseline in TNF-α (102%) ( Figure 2C), IL-6 (382%) ( Figure 2D) and IL-10 (166%) ( Figure 2E) levels at day 2. Although serum IFN-γ (73.6%), IP-10 (21.72%), and CRP (67.3%) were increased above baseline levels by the drug (day 1), the increases in IFN-γ ( Figure 2F), IP-10 ( Figure 2G) were 26- and 14-fold higher, respectively, in the norovirus-infected participant at day 2 of the trial. A 40-fold increase in CRP levels was induced by norovirus infection compared to drug alone ( Figure 2H). Notably, the peak of the CRP response was observed 24 h after the peak of proinflammatory cytokines detected in the serum. SIGLEC-1 expression on monocytes has been previously proposed as an interferon-induced biomarker of infection, vaccine response or disease activity ^16– 18^. IL-2 injection induced a small (18%) increase in sSIGLEC-1 levels above baseline. However, in line with the increased production of proinflammatory cytokines, norovirus infection induced a profound and sustained sSIGLEC-1 release (day 7 maximum, 83% increase, day 14 return to baseline) ( Figure 2I).

### Innate immune cell responses to norovirus infection

**Figure 3.**
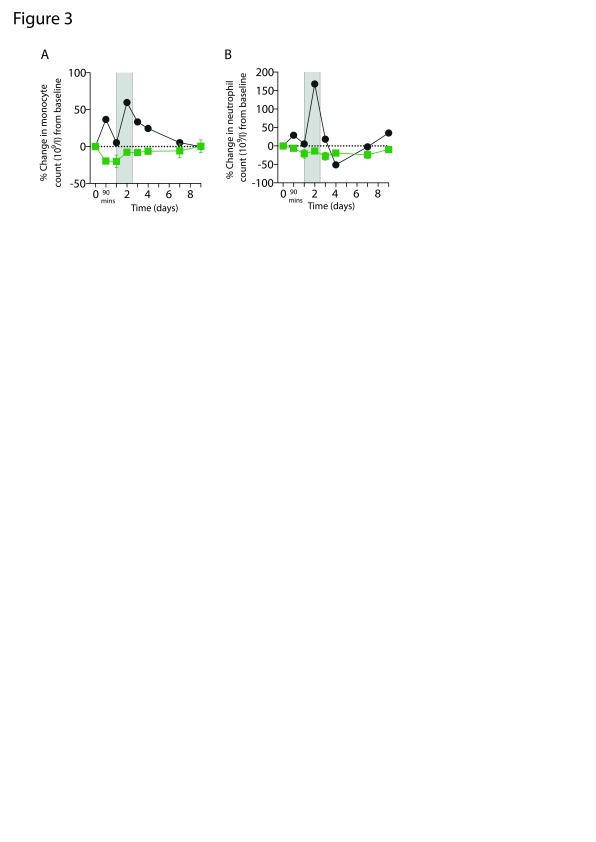
Neutrophils and monocytes are rapidly mobilised during norovirus infection. Percentage change from baseline in ( **A**) monocyte and ( **B**) neutrophil counts was determined in uninfected participants (filled green squares +/- SEM) and the norovirus-infected participant (filled black circles). Data were normalised to and expressed as percentage change from day 0 levels. The shaded area indicates the period of reported gastroenteritis.

**Figure 4.**
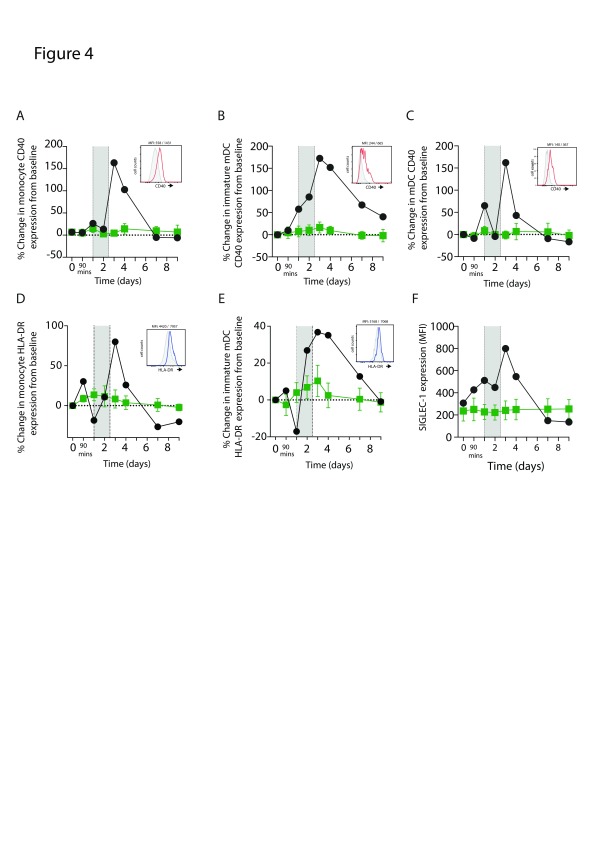
Antigen presenting cells upregulate expression of costimulatory molecules and an interferon signature biomarker. Percentage change in CD40 expression (mean fluorescent intensity, [MFI]) from baseline was determined on ( **A**) monocytes (CD14 ^++^), ( **B**) immature myeloid DC (CD11c ^+ ^CD1c ^−^lin ^−^ CD123 ^int^CD304 ^−^) or ( **C**) myeloid dendritic cells (CD1c ^+^lin ^−^CD123 ^−^CD304 ^−^) from uninfected participants (filled squares +/- SEM) or the norovirus-infected participant (filled circles). Data were normalised to and expressed as percentage change from day 0 levels. The shaded area indicates the period of reported gastroenteritis. Inset histograms show CD40 expression at pre-dosing (filled grey) or day 3 (red line) in the norovirus-infected participant for each cell subset. ( **D**) Percentage change in HLA-DR expression (MFI) from baseline on monocytes and ( **E**) immature myeloid DCs from uninfected participants (filled squares +/- SEM) or the norovirus-infected participant (filled circles). Inset histograms show HLA-DR expression for each cell type at day 3 (blue line) post-IL-2 injection compared to pre-dosing levels (filled grey) in the norovirus-infected participant. ( **F**) SIGLEC-1 expression (MFI) was determined on peripheral blood monocytes (CD14 ^++^CD16 ^lo^) in uninfected participants (filled green squares) versus the norovirus-infected participant (filled black circles). The shaded area indicates the period of reported gastroenteritis. All analyses were performed using cryopreserved PBMCs.

**Figure 5.**
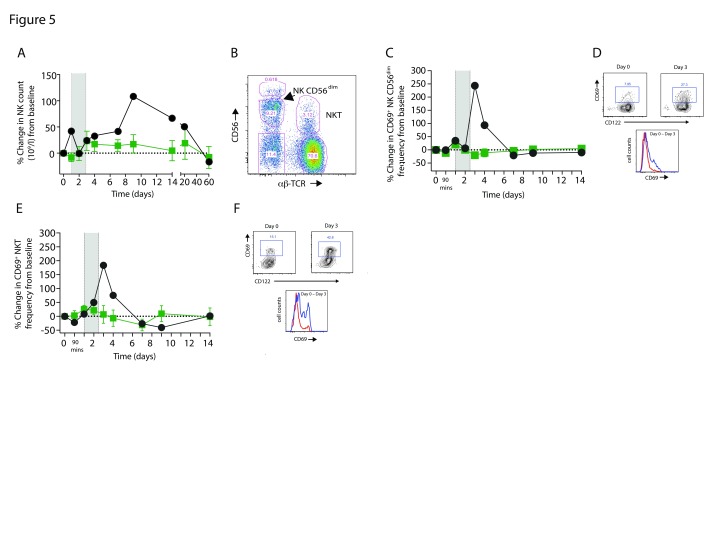
Norovirus infection induces an increase in the frequency of CD69 ^+^ NK and NKT cells. The percentage change in frequency from baseline of total NK cells was measured using multitest TBNK tubes ( **A**). Deeper analysis of blood NK, NKT and non-NK subsets was carried out using the gating strategy shown in ( **B**). The percentage change in frequency from baseline of CD69 ^+^ CD56 ^dim^ NK cells ( **C**) and CD69 ^+^ NKT cells ( **E**) was measured in uninfected participants (filled green squares +/- SEM) and the norovirus-infected participant (filled black circles). Data were normalised to and expressed as percentage change from baseline (day 0). Expression of CD69 and CD122 by αβ ^−^TCR ^− ^CD56 ^dim^ NK cells ( **D**) and αβ ^+^TCR ^+^ CD56 ^+^ NKT cells ( **F**) is shown in contour plots in the norovirus-infected participant at the indicated timepoints post-IL-2 administration. Histograms show the differential expression of CD69 on αβ ^−^TCR ^−^ CD56 ^dim^ NK cells ( **D**) and αβ ^+^TCR ^+^ CD56 ^+^ NKT cells ( **F**) at the peak of the response (day 3, blue line) versus day 0 (red line) in the norovirus-infected participant.

Norovirus infection is generally considered to be limited to the gastrointestinal tract, with little evidence of systemic dissemination of the virus in immunocompetent individuals ^5^. Norovirus RNA was not detected in circulating PBMCs from the infected participant ( Figure S1). Given the rapid proinflammatory cytokine response, we assessed whether we could detect an infection-induced immune signature in the blood. Norovirus infection dynamically increased monocyte ( Figure 3A) and neutrophil ( Figure 3B) counts on day 2, coincident with the peak of proinflammatory cytokine release. A clear induction of CD40 ( Figure 4A) and HLA-DR ( Figure 4D) was observed on monocytes from the norovirus-infected participant a day later, on day 3. CD40 and HLA-DR were also upregulated on CD1c ^−^CD304 ^−^CD123 ^int^CD11c ^+^ immature mDCs ( Figure 4B and 4E), while only CD40 expression was upregulated on myeloid dendritic cells (CD1c ^+^mDCs) ( Figure 4C). Norovirus infection induced SIGLEC-1 expression on CD14 ^++^CD16 ^lo^ classical monocytes also peaking at day 3 ( Figure 4F), one day after the peak of increased IFN-γ and IP-10 levels in the serum ( Figure 2F and 2G) and prior to the secondary peak in sSIGLEC-1 levels in the serum ( Figure 2I). Antigen-presenting cell populations in non-infected trial participants were quiescent following drug administration suggesting that the increases in CD40 and HLA-DR expression were initiated by the systemic cytokine released in response to norovirus infection. The NK cell count peaked later in the norovirus infected participant on day 9 of the trial, 7 days following infection ( Figure 5A). However, the frequency of cells expressing the activation marker CD69 was increased in the CD56 ^dim^ αβTCR ^−^ (NK CD56 ^dim^, Figure 5B–5D) and NKT cell populations ( Figure 5B, 5E and 5F) by day 3 of the trial, 1 day after infection. IL-2 administration alone had little effect on CD69 expression on CD56 ^dim^ NK and NKT cell subsets at this dose.

### Mobilisation of memory T cells by norovirus infection

**Figure 6.**
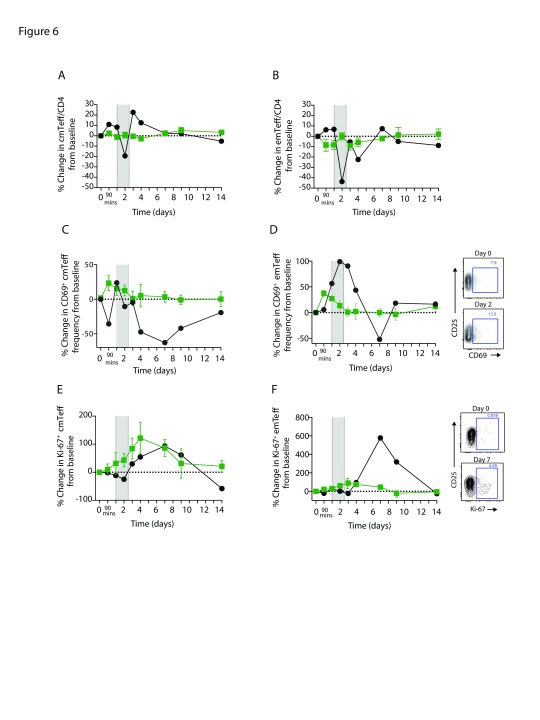
Dynamic effector memory CD4 ^+ ^T cell responses to norovirus infection. The percentage change in frequency of CD4 ^+ ^cmTeffs ( **A**), CD4 ^+ ^emTeffs ( **B**) of total CD4 ^+^ T cells from baseline levels. The percentage change in frequency of CD69 ^+^ CD4 ^+ ^cmTeffs ( **C**), CD69 ^+^ CD4 ^+ ^emTeffs ( **D**), Ki-67 ^+ ^CD4 ^+ ^cmTeffs ( **E**) and Ki-67 ^+ ^CD4 ^+ ^emTeffs ( **F**) relative to their respective baseline levels in uninfected participants (filled green square+/-SEM) and the norovirus-infected participant (filled black circles). The shaded area indicates the period of reported gastroenteritis. Contour plots showing expression of CD69 and CD25 ( **D**) and Ki-67 and CD25 ( **F**) on CD4 ^+^ emTeffs pre-IL-2 administration (day 0) and at the peak of the response in the norovirus-infected participant (day 2 or 7 respectively).

Norovirus can induce Th1 immunity and generate weak memory responses in CD4 ^+^^19^ and CD8 ^+^ T cells ^20^. However, little is known with regard to the dynamics of the adaptive T cell response directly following infection in humans. A single administration of IL-2 dynamically affects T cells, particularly Tregs, and NK CD56 ^bright^ cells ^1^. However, the frequent sampling protocol of the trial provided enough sensitivity to distinguish the systemic response to infection from drug induced changes. At day 2, CD4 ^+^ central memory (cm) Teffs (CD4 ^+^CD45RA ^−^CD62L ^hi^) and effector memory (em) Teffs (CD4 ^+^CD45RA ^−^CD62L ^lo^) decreased in frequency by 20% and 40%, respectively, compared to smaller changes induced by IL-2 administration ( Figure 6A and 6B). The behavior of these memory CD4 ^+^ T cell populations differed at day 3; CD4 ^+^ cmTeffs rebounded to a 25% increase above baseline while emTeffs returned to baseline at day 7. In humans a large proportion of tissue-resident memory Teffs express CD69, a marker of T cell receptor-mediated signaling, which distinguishes tissue-resident from circulating populations ^21^. In the circulation, CD69 expression is low ( Figure 6D, top contour panel) and is found on a small percentage of mTeffs unless there is immune perturbation. In contrast to the drug-induced 23% increase in frequency of CD69 ^+^ cmTeffs at 90 minutes ( Figure 6C), norovirus infection caused decreases of 42–62% on days 4, 7 and 9, returning toward baseline only at day 14. This prolonged decrease in the blood could be due to their homing to, and/or retention in, secondary lymphoid tissues via the CD62L homing receptor. A decline in frequency of cmTeffs in cell cycle (Ki-67 ^+^) at day 2 was also observed in the norovirus-infected participant ( Figure 6E). However, the later recovery and increase in frequency of Ki-67 ^+^ cmTeffs peaking at day 4 was similar in the drug-treated group and the norovirus-infected participant. In contrast to the CD69 ^+^ cmTeff response, norovirus infection doubled the proportion of CD69 ^+^ emTeffs in the circulation during acute gastroenteritis (day 2) ( Figure 6D) and induced a 577% increase in frequency of Ki-67 ^+^ emTeff at day 7 ( Figure 6F).

**Figure 7.**
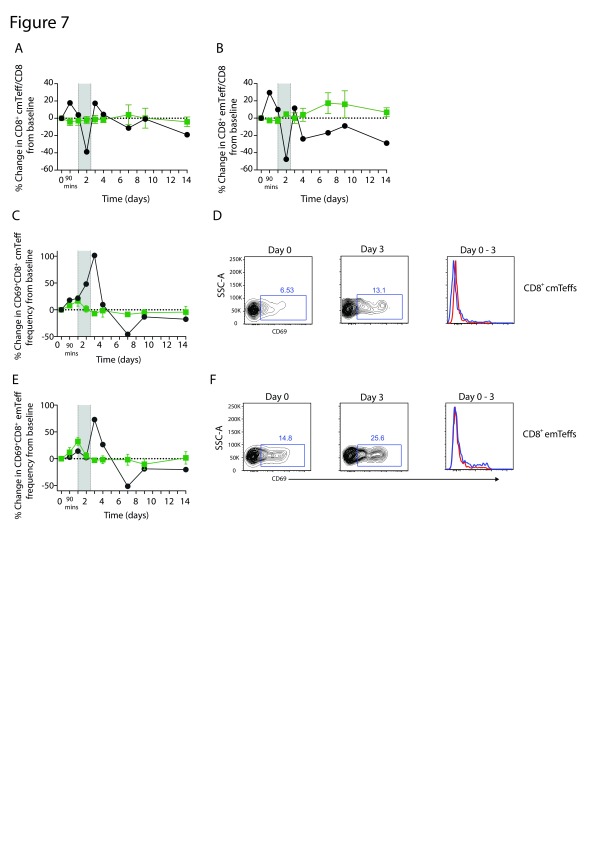
Temporal changes in frequency and CD69 expression in memory CD8 ^+^ T cell subsets immediately following norovirus infection. The percentage change in frequency of CD8 ^+^ cmTeffs ( **A**), CD8 ^+ ^emTeffs ( **B**) of total CD8 ^+^ T cells from baseline levels. The percentage change in frequency of CD69 ^+^ CD8 ^+^ cmTeffs ( **C**) CD69 ^+^ emTeffs ( **E**) relative to baseline levels in uninfected participants (filled green square+/-SEM) and the norovirus-infected participant (filled black circles). The shaded area indicates the period of reported gastroenteritis. Contour plots showing expression of CD69 in the CD8 ^+^ cmTeff ( **D**) and CD8 ^+^ emTeff ( **F**) populations, pre-IL-2 administration (day 0) and at the peak of the response in the norovirus-infected participant (day 3). Histograms show the differential expression of CD69 on cmCD8 ^+^ T cells ( **D**) and emCD8 ^+^ T cells ( **F**) at the peak of the response (day 3, blue line) versus day 0 (red line) in the norovirus-infected participant.

**Figure 8.**
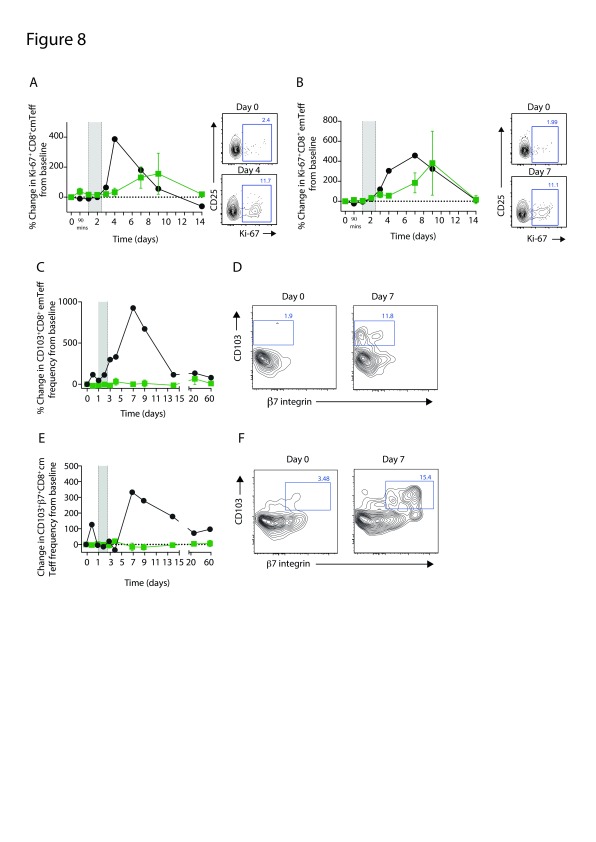
The emergence of memory CD8 ^+ ^Teffs in cell cycle and with mucosal homing capacity post-norovirus infection. The percentage change in frequency of Ki-67 ^+^ CD8 ^+^ cmTeffs ( **A**) and Ki-67 ^+^ CD8 ^+ ^emTeffs ( **B**). Contour plots for each cell subset show Ki-67 and CD25 expression pre-IL-2 administration and at the peak of the response in the norovirus-infected participant. Timecourse analysis of the frequency of CD103 ^+^CD45RA ^−^CD27 ^−^ CD8 ^+^ emTeffs of total CD8 ^+^ T cells ( **C**). Contour plots showing the frequency of CD103 ^+^β7integrin ^−/lo^CD45RA ^−^CD27 ^−^ CD8 ^+^ emTeffs at day 0 and day 7 post-IL-2 ( **D**). ( **E**) The change in frequency of CD103 ^+^β7integrin ^+^CD45RA ^−^CD27 ^+^ CD8 ^+^ cmTeffs of total CD8 ^+^ T cells relative to baseline levels. ( **F**) Contour plots showing the frequency of CD103 ^+^β7 integrin ^+^CD45RA ^−^CD27 ^+^ CD8 ^+^ cmTeffs at day 0 and day 7 post-IL-2. ( **A**, **B**, **C** and **E**) data were normalised to and expressed as percentage change from baseline (day 0) in uninfected participants (filled squares +/- SEM) or the norovirus-infected participant (filled circles). Analyses were performed using cryopreserved PBMCs. The shaded area indicates the period of reported gastroenteritis.

Norovirus infection also induced dynamic changes in the CD8 ^+^ T cell population while IL-2 administration alone had minimal effects. The frequency of both CD8 ^+ ^cmTeffs (CD8 ^+^CD45RA ^−^CD62L ^hi^) and CD8 ^+ ^emTeffs (CD8 ^+^CD45RA ^−^CD62L ^lo^) decreased at day 2 in the blood by approximately 40%, recovering to around baseline levels by days 4–9 ( Figure 7A and 7B). Frequencies of CD69 ^+^CD8 ^+^ cmTeffs and CD69 ^+^CD8 ^+^ emTeffs both showed distinct peaks, ranging between 50% and 100% changes at day 3 ( Figure 7C–F). Norovirus infection also induced a 387% increase in frequency of Ki-67 ^+^CD8 ^+^ cmTeffs at day 4 ( Figure 8A) and a 457% increase in Ki-67 ^+^CD8 ^+^ emTeffs at day 7 ( Figure 8B). These data also revealed the dynamic nature of the CD8 ^+^ mTeff compartment itself, with one control participant showing increases in frequency of 662% and 1614% in Ki-67 ^+^ cmCD8 ^+^ Teff and Ki-67 ^+^ emCD8 ^+^ Teff, respectively, at day 9 post-treatment ( Figure 8A and 8B).

Mouse models suggest that norovirus infection and the specific immune response is largely confined to the intestinal mucosa ^22^ and associated Peyer’s patches ^23^. The increase in frequency of CD69 ^+^CD8 ^+^ Teffs suggested the release and expansion of tissue-resident T cells following infection. We therefore assessed αE (CD103) and β7 integrin expression on CD8 ^+^ mTeffs as the heterodimer αEβ7 is expressed on a large proportion of intestinal and tissue resident lymphocyte populations ^24^. Populations of circulating CD8 ^+^ emTeffs (CD8 ^+^CD45RA ^−^CD27 ^lo^) expressing CD103 ( Figure 8C and 8D) and CD103 ^+^β7 ^+^CD8 ^+^ cmTeffs (CD8 ^+^CD45RA ^−^CD27 ^hi^) ( Figure 8E and 8F) were induced in the norovirus-infected participant to 926% and 332% above baseline levels, respectively, both peaking at day 7. 

### Norovirus infection induces a profound counter regulatory response

**Figure 9.**
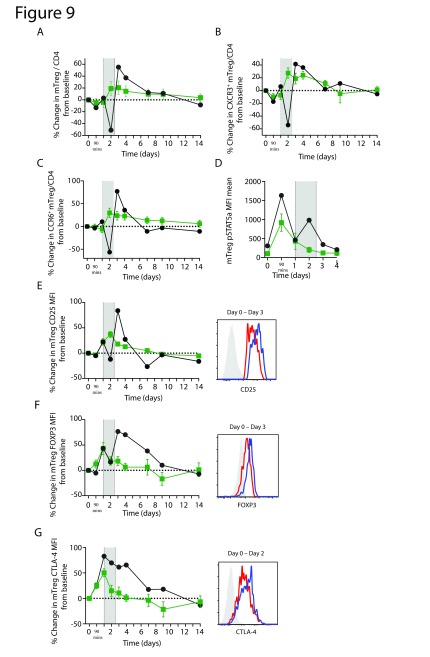
A counter-regulatory response to norovirus infection is coincident with effector cell mobilisation. The percentage change in frequency of mTregs ( **A**), CXCR3 ^+^ mTregs ( **B**) and CCR6 ^+ ^mTregs ( **C**) of total CD4 ^+^ T cells in the norovirus-infected and uninfected participants. Phosphorylation of STAT5a (MFI) in mTregs in the norovirus-infected participant and controls (filled squares +/- SD) ( **D**). The percentage change in CD25 ( **E**), FOXP3 ( **F**) and CTLA-4 ( **G**) expression (MFI) relative to baseline on mTregs in controls or the norovirus-infected participant. Data were normalised to and expressed as percentage change from baseline (day 0) and measured in uninfected participants (filled green squares +/-SEM, n=5 apart from 9B and 9C where n=3) and the norovirus-infected (filled black circles) participant. The shaded area indicates the period of reported gastroenteritis. Histograms show the differential expression of CD25 ( **E**), Foxp3 ( **F**) and CTLA-4 ( **G**) at the peak of the response (day 3: CD25, FOXP3 and day 2: CTLA-4, blue line) versus day 0 pre-IL-2 (red line) and the shaded grey histogram is expression of CD25, CTLA-4 and FOXP3 in naïve CD4 ^+^ Teffs at day 0 in the norovirus-infected participant, as a negative control for staining.

Regulatory cells are critical to host tissue integrity during infection ^25^. Despite the infection being contained within the gastrointestinal tract, along with the other systemic effects observed on innate and adaptive effector immune cell subsets, norovirus infection induced profound phenotypic changes in the Treg compartment in the blood. Norovirus infection induced a 9-fold larger alteration in trafficking of mTregs (51% reduction) from the blood at day 2 compared to the control group at 90 minutes after drug administration (5% reduction) and the participant’s 90 minute post-treatment levels (13% reduction) ( Figure 9A). Notably, these alterations broadly affected the Treg compartment, including the tissue homing CXCR3 ^+^ and CCR6 ^+^ mTreg subsets ( Figure 9B and 9C). The subsequent expansion of mTregs in blood was also larger in the norovirus-infected participant at day 3 compared to the drug-induced expansion (50% versus 20% respectively) before returning to baseline by day 7–14. In contrast, norovirus infection did not increase the frequency of naïve Tregs over the observed IL-2-induced increase ( Figure S2A). Norovirus infection induced a second increase of STAT5a phosphorylation in mTregs ( Figure 9D and Figure S3A) and CD4 ^+^ mTeffs ( Figure S3A) that was coincident with the release of cytokines, including IL-2, at day 2 and was not observed in control non-infected participants ( Figure S3B). Norovirus infection also induced greater increases of mTreg CD25 ( Figure 9E), FOXP3 ( Figure 9F) and CTLA-4 expression ( Figure 9G) and compared to IL-2 treatment, an effect also apparent in naïve Tregs albeit at slightly lower levels ( Figure S2B–D).

## Discussion

Frequent sampling, initially at daily intervals, revealed the broad and highly dynamic systemic response induced by norovirus infection. An acute type-1 proinflammatory cytokine release, presumably by innate lymphocytes in the gastrointestinal mucosal tissues, was followed by a systemic activation and mobilisation of cells of both the innate and adaptive immune systems. Of particular interest and novelty was the immediate and prolonged Treg response induced by norovirus infection. The adaptive clinical trial DILT1D was primarily designed to examine the Treg response to a single dose of IL-2 and we noted the similarity between the systemic response of Tregs to the drug to that induced by norovirus infection. Both perturbations induced immediate trafficking of Tregs followed by expansion and upregulation of key effector molecules, CD25, CTLA-4 and FOXP3.

The symptoms and the induction of norovirus-specific immune responses support norovirus as the most likely clinical cause of the infection in our study. However, without detection of viral RNA, we were unable to definitively show that norovirus was the causative organism or to identify the exact strain of norovirus in the infected participant. In the absence of other participants developing norovirus infection during the study we describe an n-of-1 case study in a participant who developed the norovirus infection proximal to drug administration. We are confident that the five uninfected participants adequately controlled for the influence of the administration of IL-2 on the immune phenotypes induced by norovirus infection. Although we can not exclude the possibility that some of the responses that we observe were not enhanced by the administration of IL-2.

Similar to norovirus challenge infections ^10, 26^, the natural infection reported here transiently increased serum IFN-γ, IL-2, IL-6 and TNF-α concentrations. The increases in TNF-α and in monocyte SIGLEC-1 expression from 90 minutes onwards (in contrast to the five non-infected participants who received the same dose of IL-2 and were not infected with norovirus) are supportive of the norovirus infection being initiated near the time of drug administration. We also note how prolonged the sSIGLEC-1 response was compared to monocyte surface SIGLEC-1 expression. Monocyte/macrophage expression of SIGLEC-1 can be considered as a cell-specific biomarker of immunologically active cells ^27^ and we attribute this to prolonged activation of monocytes in the gut and sustained release of sSIGLEC-1. Therefore, we propose sSIGLEC-1 is a good candidate as a relatively long-lived marker of interferon release and inflammation.

Robust innate cell activation and modulation of antigen presenting cell populations was a feature of the cellular response following infection. Activation markers HLA-DR and CD40 were upregulated on monocytes and dendritic cell subsets one day after the peak of cytokine in the serum. Norovirus capsid protein can be detected in cells of the immune system such as T cells, macrophages and dendritic cells ^28, 29^ but appears to actively replicate in enterocytes *in vivo* and *in vitro* ^3, 28, 30^. Given the homogeneous upregulation of HLA-DR and CD40 on dendritic cells and monocytes, and, as was the case with the upregulation of SIGLEC-1 expression on monocytes, the increased expression most likely reflects a response to systemic inflammatory cytokine release ^31, 32^. The frequency of CD69 ^+^CD56 ^dim^NK cells, a critical anti-viral effector population, acutely increased during infection, and may also have been activated indirectly in response to interferon release ^33^. In mouse models, NKT cells are a key regulator of microbial homeostasis ^34^ and could therefore have upregulated CD69 directly in response to norovirus in the gut. However cytokines, including IL-12, can also activate NKT cells and upregulate CD69 expression ^35^. We therefore conclude that the majority of the innate cell activation signature observed in the blood was in response to the systemic cytokine release from mucosal immune cells exposed directly to the viral infection.

Despite the limitation of our study to individuals with type 1 diabetes participating in a clinical trial, detailed insights into the biology of Tregs in infection were achieved. Tregs are a key immune cell subset critical to maintaining immune tolerance, and have been considered to be defective in type 1 diabetes ^36^. The increase in Treg frequency following norovirus infection was consistent with observations from studies in non-diabetic individuals with acute and chronic viral infections, malaria, and bacterial or fungal sepsis ^37– 43^. However in the absence of healthy controls and additional T1D patients with or without norovirus infection we cannot determine whether the regulatory or effector responses to infection are optimal in our participants with diabetes. Modelling disease outcomes in mice has highlighted the complex inter-relationship between effector function and immune regulation by Tregs in response to infection. Rather than suppressing immune responses, Tregs were shown to coordinate immunity at the mucosal site of infection ^44^ and facilitate the generation of memory CD8 ^+^ T cells ^45, 46^ in acute viral infections models. The disease course was of normal duration in the affected participant and, clinically, Proleukin administration did not affect the response to norovirus.

The lack of pre-infection baseline data from participants or sampling close to disease diagnosis in studies of acute infection ^38, 39, 43^ appears to have precluded the identification of the trafficking of Tregs from the blood in response to the acute increase in cytokine concentrations, including IL-2. Even in a very recent study of the immune responses of humans to influenza vaccination, Treg trafficking was not reported, although they did identify a novel initial “lymphoid stress signature”, which they proposed requires further study ^47^. Increases in serum levels of the CXCR3 ligand IP-10 (CXCL10) associated with IL-2 administration were much greater in norovirus infection and coincident with a reduction in frequency of CXCR3 ^+^ mTregs in the blood. CXCR3 ^+^ Tregs are thought to home to sites of type-1 inflammation, and IFN-γ signaling in Tregs promotes CXCR3 ^+^ expression and control of Th1 immune responses in mice ^48^. The dramatic decline and increased rebound in the frequency of CCR6 ^+^ mTregs in the blood, and IL-10 production following norovirus infection may reflect the compartmentalised gastrointestinal nature of the infection itself ^4^. In support of this possibility, increased serum CCL20 levels and an increased frequency of CCR6 ^+^ Tregs has been identified in patients with *H. pylori* ^49^. CCR6 ^+^ mTregs induced by IL-2 administration or infection may traffic to mucosal tissues including to the gut in response to an increase in the expression of the chemokine CCL20 by the mucosa, owing to release of proinflammatory cytokines ^50, 51^.

Norovirus infection also induces a counter-regulatory response that is greater than that induced by subcutaneous IL-2 administration, with a larger expansion of Tregs that have augmented expression of molecules associated with increased Treg function: CTLA-4, FOXP3, CD25 and increases in serum IL-10. The production of IL-10, critical for mucosal integrity after norovirus infection in mice ^52^, coincides with the peaks of endogenous IL-2 and the proinflammatory cytokines IL-6 and IFN-γ during acute gastroenteritis at day 2. The peak of serum cytokines also corresponded with a secondary peak of pSTAT5 expression that was predominant in Tregs that was most likely, but not exclusively, induced by endogenous IL-2. Here we link the increase in Treg frequency and Treg CD25, CTLA-4 and FOXP3 expression to the infection-induced release of IL-2, detectable in the serum. The Treg phenotypes observed with infection were remarkably similar to those induced by drug alone. Although norovirus infection-induced IL-2 levels in the blood were comparable to those observed at 90 minutes following IL-2 administration, we speculate that local IL-2 levels in the infected tissue would be considerably higher. In the absence of healthy controls with or without norovirus infection we can not determine whether the regulatory or effector responses to infection are optimal in our participants with diabetes. However, our data are consistent with mouse models ^53^ illustrating a remarkable simultaneous and coordinated induction of regulatory and effector / inflammatory immune cell activation. Our data also highlight the role of key Treg molecules, CTLA-4, CD25, IL-10 and IL-2, genetically associated with the development of type 1 diabetes, in the response to infection.

In summary, norovirus infection induces a systemic immune signature in the innate and adaptive immune systems. Counter-regulatory mechanisms are instructed early in the response to infection to temper collateral tissue damage, regulatory mechanisms that are also induced during IL-2 therapy.

## Data availability

The data referenced by this article are under copyright with the following copyright statement: Copyright: © 2017 Cutler AJ et al.

The data cannot be anonymised sufficiently to be able to put it into the public domain without the risk of participant identification. Participant level data is available on request through the University of Cambridge institutional repository. All necessary information on how to apply for access to the data, and the conditions under which access will be granted, are available by following the link provided:

https://doi.org/10.17863/CAM.7926 ^54^

## Consent

Individuals interested in enrolling in DILT1D were provided with a patient information sheet and an informed consent form to review and were given a minimum of 24 h to consider the information provided. Interested potential participants were then invited to attend a visit where the Chief Investigator or delegate discussed the study with the participant, who then provided written informed consent before undergoing screening and any other trial-related procedures. Participants consented to the publication of the anonymised results from the study.

## Supplementary materials

**Supplementary Figure 1. No detectable norovirus RNA in PBMC during symptomatic or asymptomatic period.** ( **A**) Real time RT-PCR quantification of norovirus GII standard plasmids titrated from 1×10 ^8^ to 1×10 ^1^ copies. ( **B**) Real time qRT-PCR analysis of RNA derived from PBMC taken from all study timepoints for the infected participant +/- reverse transcriptase.

**Supplementary Figure 2.** **Naïve Tregs increase expression of effector molecules following norovirus infection.** The percentage change in frequency of naive Tregs ( **A**) of total CD4 ^+^ T cells in the norovirus-infected participant and uninfected participants from baseline levels (day 0). The percentage change in CD25 ( **B**), FOXP3 ( **C**) and CTLA-4 ( **D**) expression (mean fluorescent intensity, [MFI]) relative to baseline on nTregs in controls or the norovirus-infected participant. Data were normalised to and expressed as percentage change from baseline (day 0) and measured in uninfected participants (filled green squares +/-SEM) and the norovirus-infected (filled black circles) participant. The shaded area indicates the period of reported gastroenteritis.

**Supplementary Figure 3. pSTAT5a in mTregs and mTeffs in an uninfected participant and the norovirus-infected participant.** Pseudocolour flow cytometry plots showing phosphorylation of STAT5a in CD3 ^+^CD45RA ^−^ mTeffs (FOXP3 ^-^) and mTregs (FOXP3 ^+^) in the norovirus-infected participant (IL-2 dose : 0.433 × 10 ^6^ IU/m ^2^) ( **A**) and a representative uninfected participant (IL-2 dose : 0.408 × 10 ^6^ IU/m ^2^) ( **B**) pre- administration (Day 0) and at the indicated timepoints post-IL-2 administration.

**Supplementary Figure 4. Figure 4A–F gating strategy.**

**Supplementary Figure 5. Figures 6, 7, 8A, 8B, 9, and Supplementary Figure 2 gating strategy.**

**Supplementary Figure 6. Figures 8C, 8D, 8E and 8F gating strategy.**

**Supplementary Figure 7. Figure 9D gating strategy.**

**Supplementary Table 1. Antibody panels used for FACS staining.** Antibody clones, fluorochromes and suppliers of specific antibodies used for staining surface molecules on whole blood (panels 1–4), intracellular and surface molecules on whole blood (panel 5 and 6) and surface molecules on previously cyropreserved PBMC (panels 7–9).
