# Supplementary figures and images for: Capturing the systemic immune signature of a norovirus infection: an n-of-1 case study within a clinical trial
Source: Wellcome Open Res. 2017 Oct 5;2:28. Originally published 2017 Apr 18. [Version 3] doi: 10.12688/wellcomeopenres.11300.3 (PMC5531165; doi:10.12688/wellcomeopenres.11300.3)

# Supplementary figure 1

## A

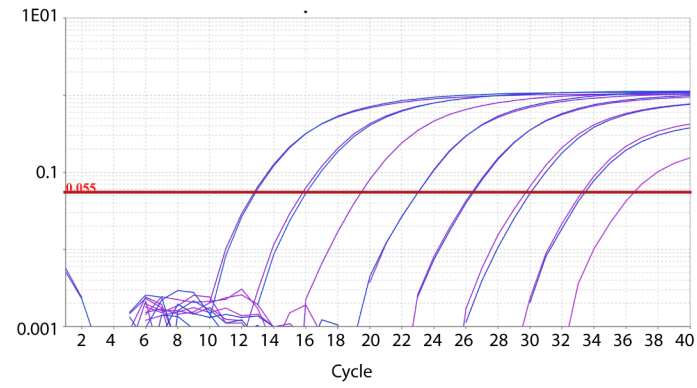

## B

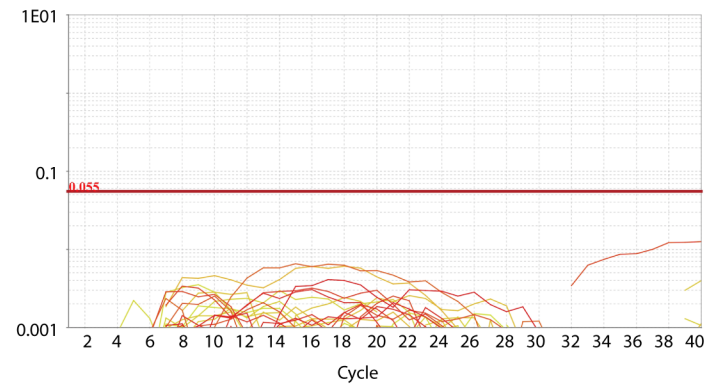

Supplement: Supplementary file 1 [file wellcomeopenres-2-13931-s0000.tgz › b1cfe105-a0ff-402a-b25d-7d8bff7a91a5.pdf]

Supplementary Figure 2

A

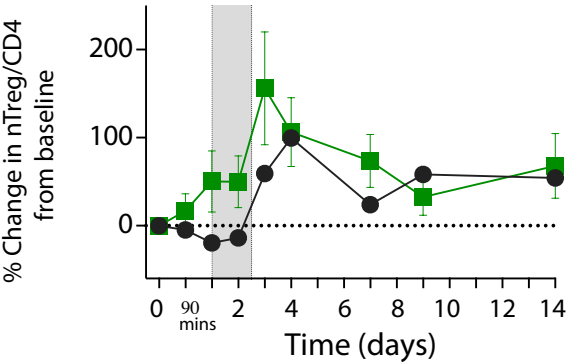

B

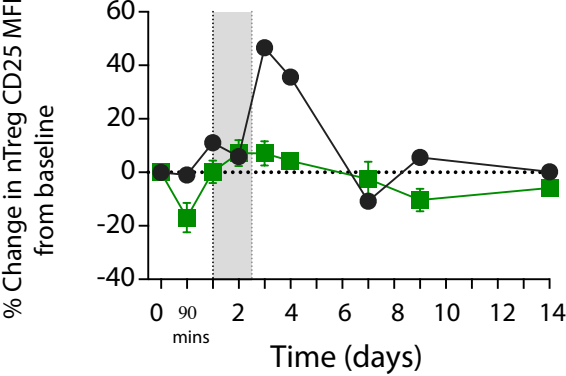

C

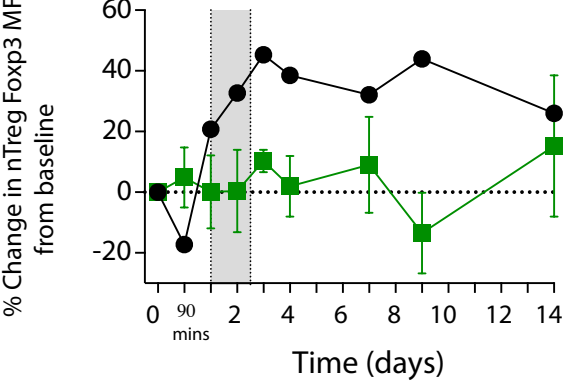

D

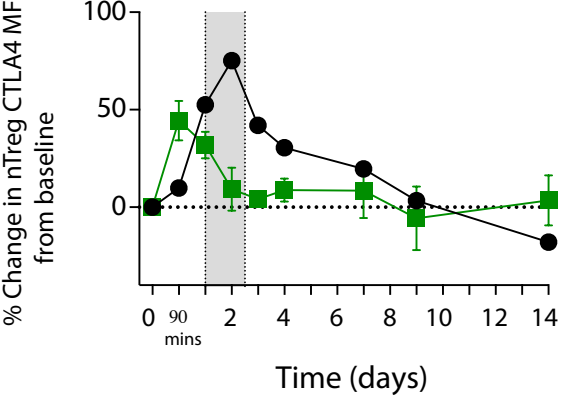

Supplement: Supplementary file 2 [file wellcomeopenres-2-13931-s0001.tgz › 07f9ef82-8a94-48d3-8a3f-0576a3d2a9f0.pdf]

Supplementary figure 3

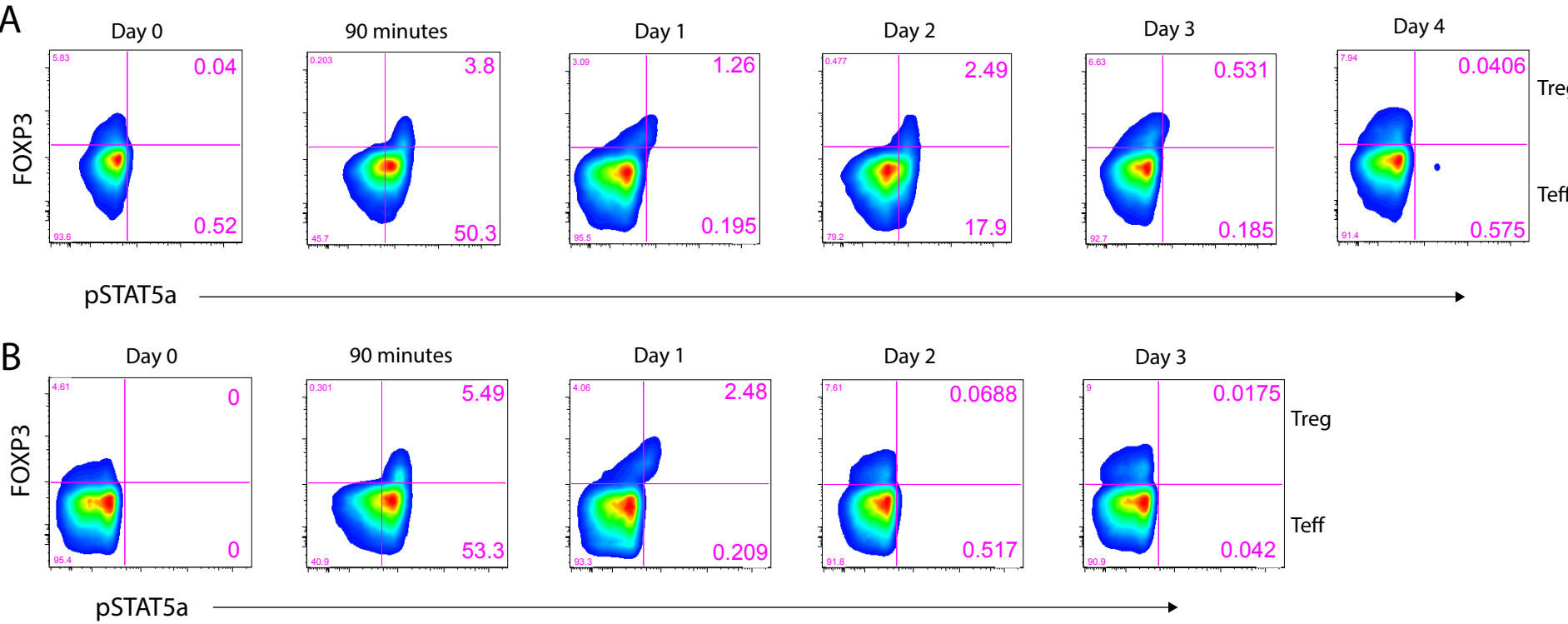

Supplement: Supplementary file 3 [file wellcomeopenres-2-13931-s0002.tgz › c9383faf-fb04-4ca4-991b-f45497a112bc.pdf]

Figure 4A - E gating strategy

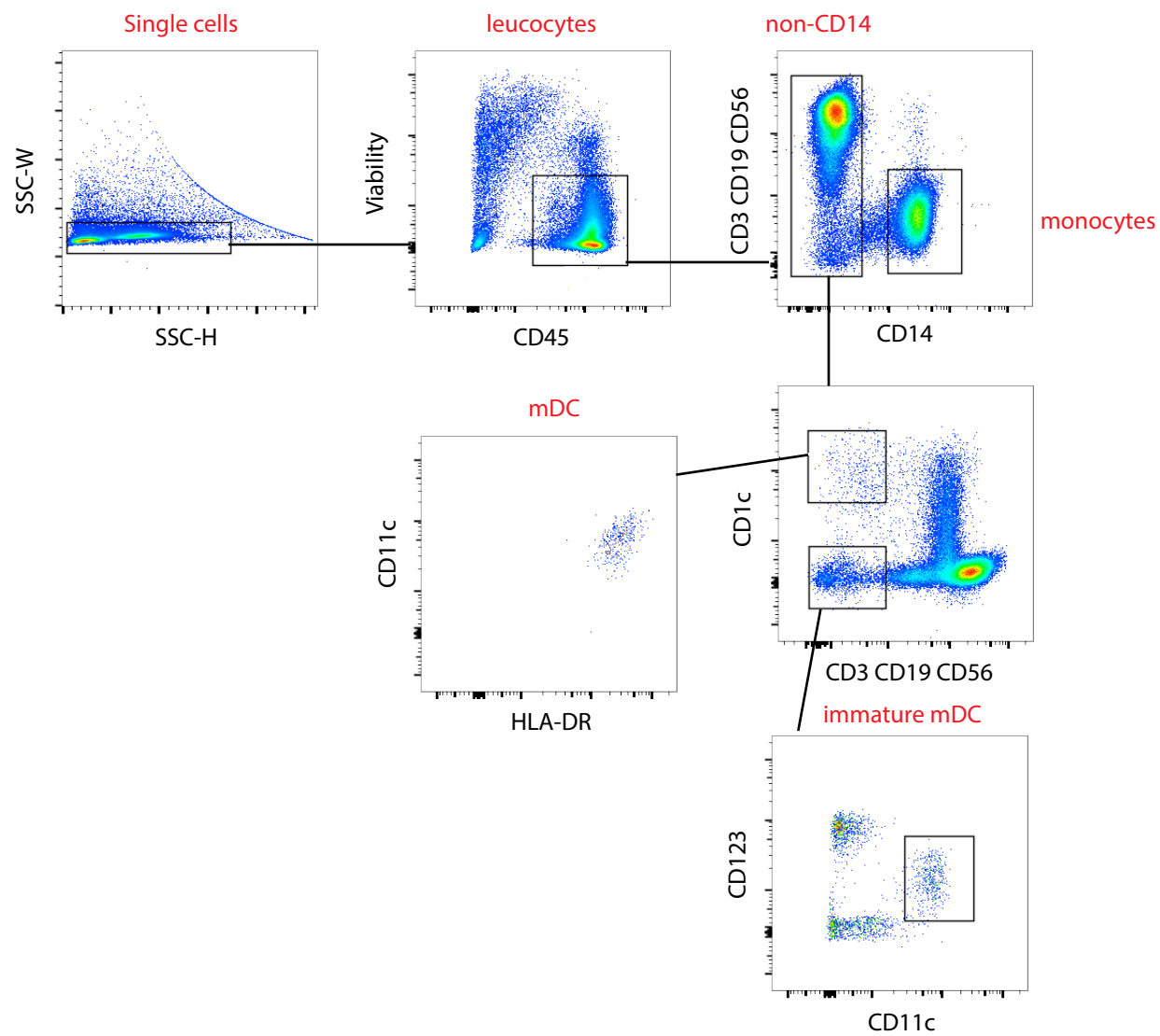

Figure 4F gating strategy

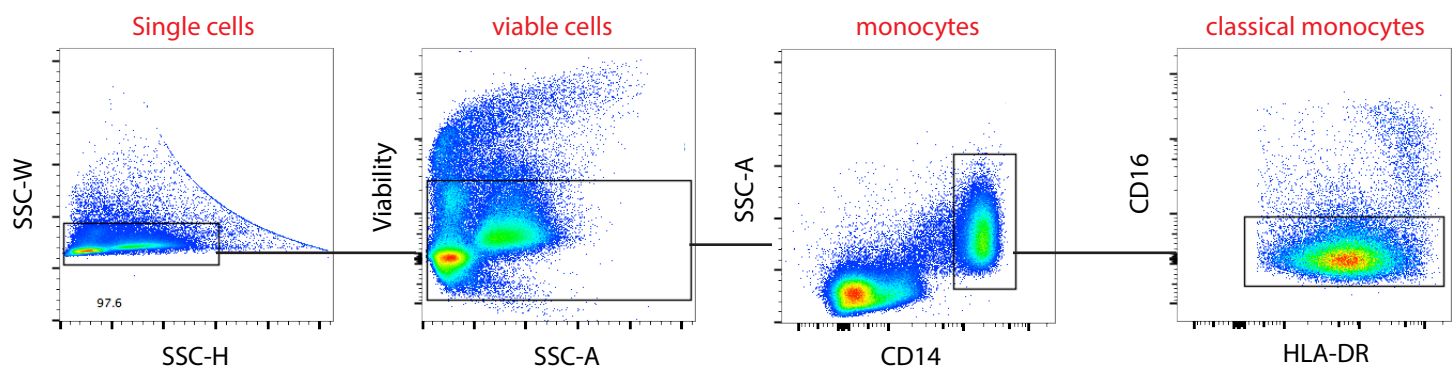

Supplement: Supplementary file 4 [file wellcomeopenres-2-13931-s0003.tgz › 06d9042e-6100-4308-afe7-108f9312aba2.pdf]

Figures 6, 7, 9 and supplementary figure 2 gating strategy

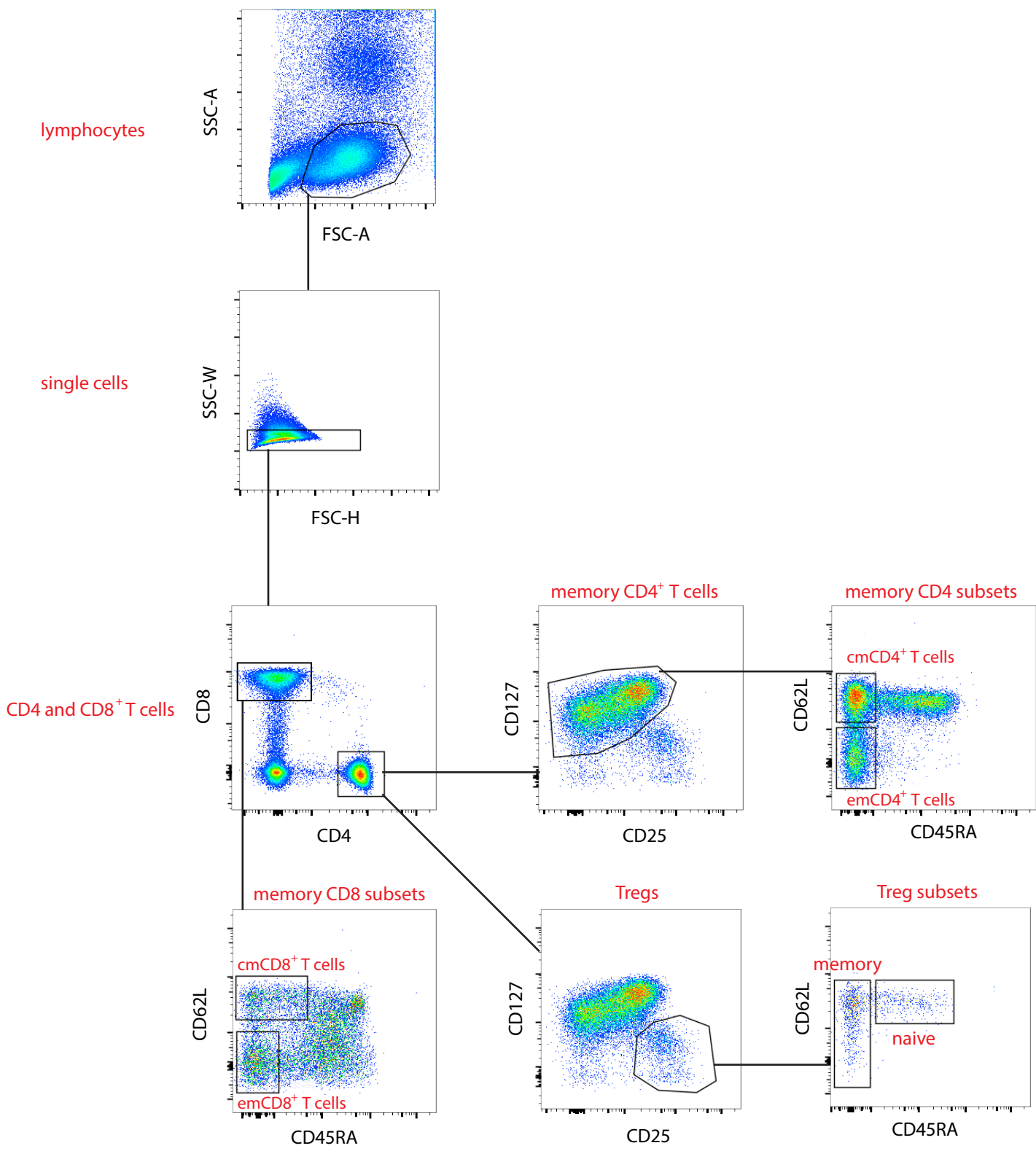

Supplement: Supplementary file 5 [file wellcomeopenres-2-13931-s0004.tgz › 4b54fac4-a352-4374-9c7b-09e97bc5976d.pdf]

Figure 8 gating strategy

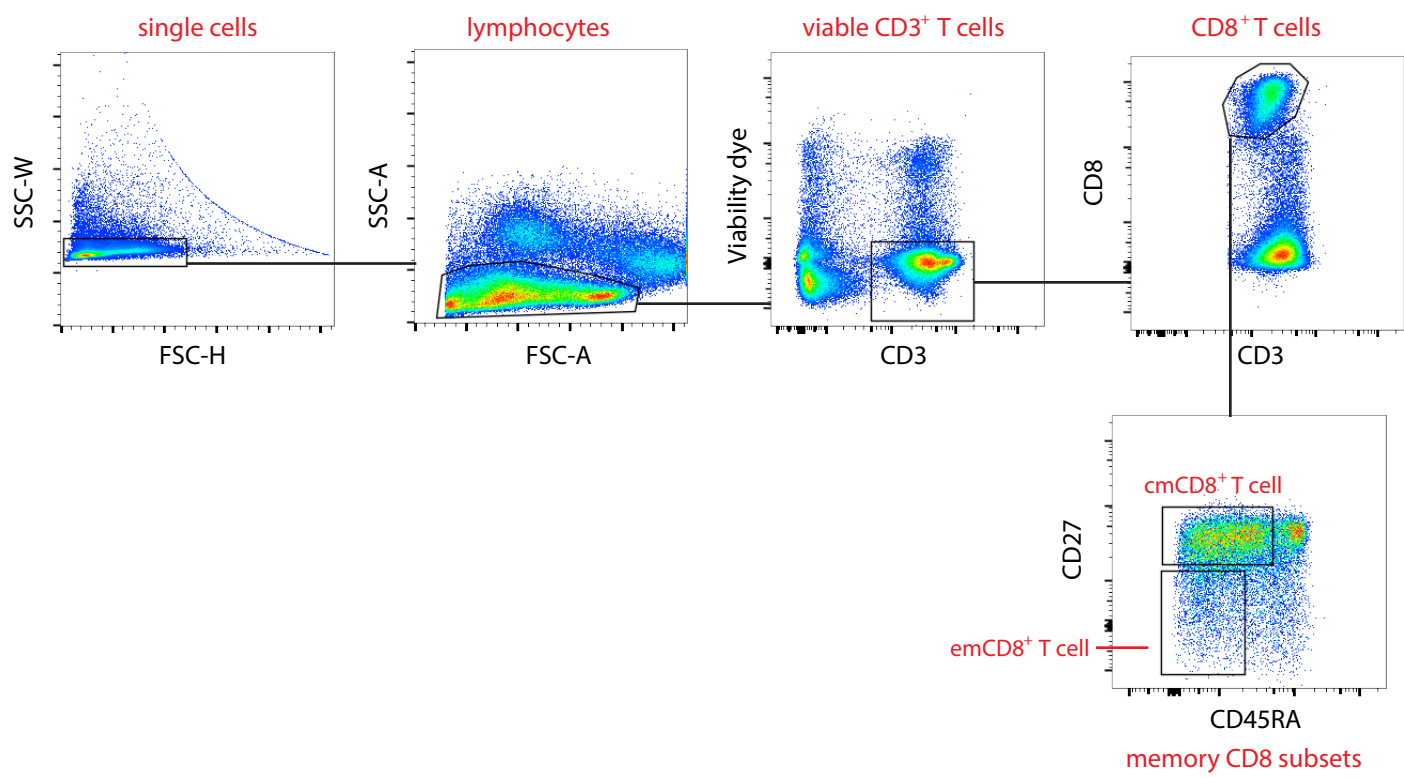

Supplement: Supplementary file 6 [file wellcomeopenres-2-13931-s0005.tgz › a081fdee-4d38-4528-946b-178f3c2209cd.pdf]

Figure 9D gating strategy

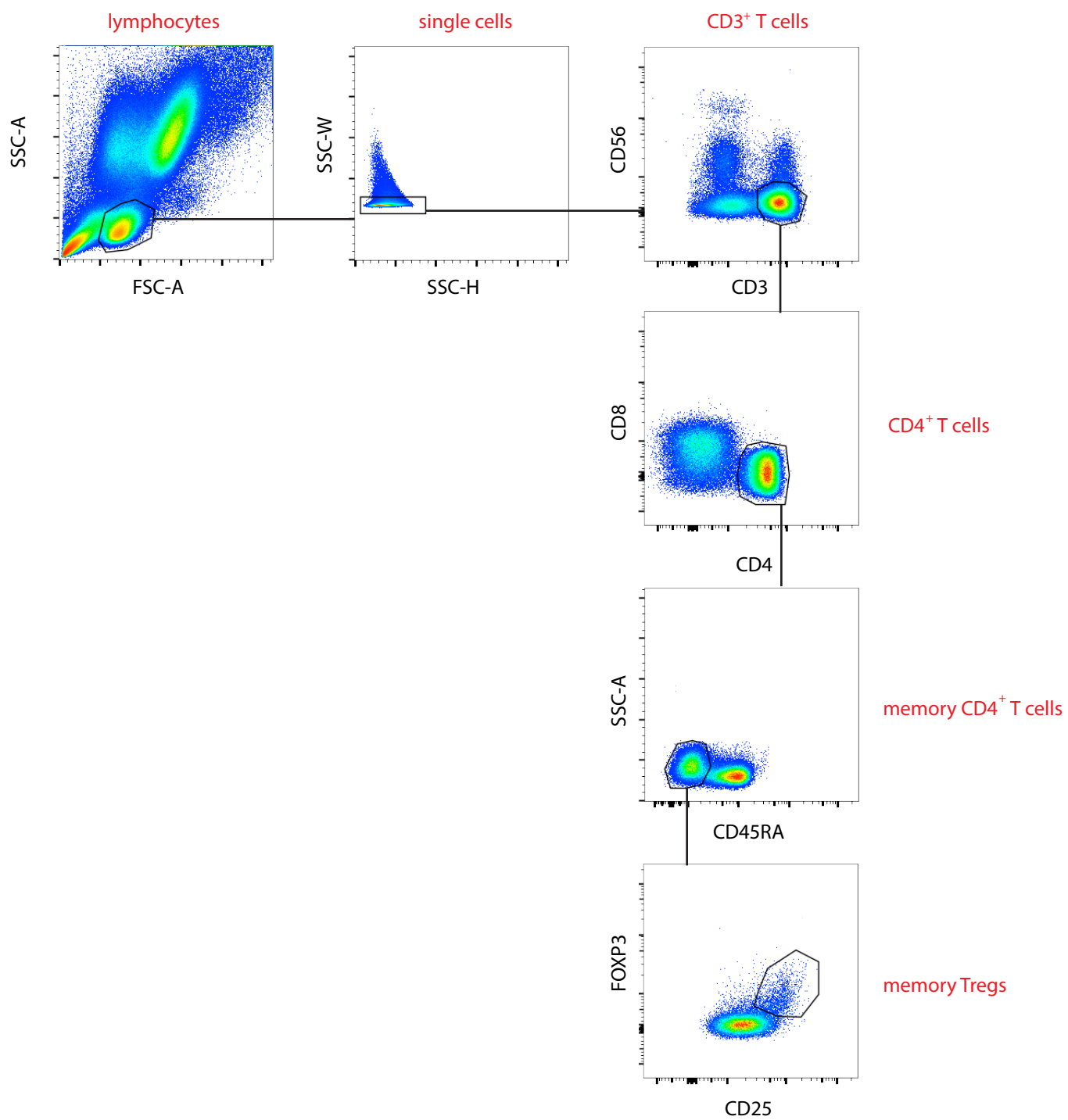

Supplement: Supplementary file 7 [file wellcomeopenres-2-13931-s0006.tgz › 54084014-c6c8-414a-b63b-957f9dbb583b.pdf]
