# Supplementary material for: Capturing the systemic immune signature of a norovirus infection: an n-of-1 case study within a clinical trial
Source: Wellcome Open Res. 2017 Oct 5;2:28. Originally published 2017 Apr 18. [Version 3] doi: 10.12688/wellcomeopenres.11300.3 (PMC5531165; doi:10.12688/wellcomeopenres.11300.3)
Supplement: Supplementary file 8 [file wellcomeopenres-2-13931-s0007.tgz › db24a570-f75f-486c-be5a-de5cbbdda4f8.pdf]

	Clone(s)	Species	Panel 1	Panel 2	Panel 3	Panel 4	Panel 5	Panel 6	Panel 7	Panel 8	Panel 9	Supplier	Catalogue no.
CD25	2A3 + M-A251	Mouse	APC	APC	APC	APC	APC	APC	APC		PE	BD	340907<br>555434<br>555432
CD4	RPA-T4	Mouse	AF700	AF700	AF700	AF700	AF700	AF700	AF700		AF700	Biolegend	300526
CD45RA	HI100	Mouse	BV785	BV785	BV785	BV785	BV785	Pacific blue	BV785		BV785	Biolegend	304140<br>304123
CD127	eBioRDR5	Mouse	PE/Cy7	PE/Cy7	PE/Cy7		PE/Cy7		PE/Cy7		PE/Cy7	eBioscience	25-1278-42
CXCR3	GO25H7	Mouse	PerCP/Cy5.5	PerCP/Cy5.5	PerCP/Cy5.5							Biolegend	353714
CCR4	2G12	Mouse			BV421							Biolegend	131218
HLA-DR	L243	Mouse	Pacific blue	Pacific blue					FITC			Biolegend	307633<br>307604
CD14	ME52	Mouse							BV421	BV785		Biolegend	301830<br>301840
CCR6	GO34E3	Mouse	AF488	AF488	AF488				PerCP/Cy5.5			Biolegend	353414<br>353406
CTLA4	BN13	Mouse	PE				PE					BD	555853
CD69	FN50	Mouse		PE		PerCP/Cy5.5						Biolegend	310906<br>310926
CCR10	FAB3478P	Rat			PE							R&D	FAB3478P
CD8	RPA-T8	Mouse	APC/Cy7	APC/Cy7	APC/Cy7	APC/Cy7	APC/Cy7	BV605	PE-Dazzle594		PE-<br>Dazzle594	Biolegend	301016<br>301040<br>301058
CD62L	DREG-56	Mouse	BV605	BV605	BV605		BV605					Biolegend	304834
CD56	HCD56	Mouse				BV421		PE/Cy7		PE		Biolegend	318328<br>318306<br>318318
αβTCR	IP26	Mouse				FITC						Biolegend	306706
CD122	TU27 & Mik-β3	Mouse				PE						Biolegend<br>BD	339006<br>554525
CD161	HP-3G10	Mouse				BV605						Biolegend	339916
Ki-67	B56	Mouse					PerCP/Cy5.5					BD	561284
FOXP3	259D	Mouse					Pacific blue	PE				Biolegend	320216<br>320208
pSTAT5a	47/Stat5(pY694)	Mouse						AF488				BD	562075
CD3	UCHT1	Mouse						PerCp/Cy5.5	BV510	PE		Biolegend	300430<br>300448<br>300408
CD3	UCHT1	Mouse									BUV395	BD	563546
viability									eFluor780	eFluor780	eFluor780	eBioscience	65-0865
SIGLEC-1	7-239	Mouse							PE			Biolegend	346004
CD27	M-T271	Mouse							BV605		BV605	BD	740398
CD16	3G8	Mouse							BV711			Biolegend	302044
CD11a	HI111	Mouse									FITC	Biolegend	301206
CD49d	9F10	Mouse									BV510	Biolegend	304318
β7<br>integrin	FIB504	Mouse									APC	Biolegend	321208
CD103	Ber-Act8	Mouse									BV421	Biolegend	350214
CD45	HI30	Mouse								UV395		BD	563792
CD25	BC96	Mouse								AF700		Biolegend	302622
CD19	HIB19	Mouse								PE		Biolegend	302208
CD40	5C3	Mouse								PE/Cy7		Biolegend	334322
CD123	6H6	Mouse								FITC		Biolegend	306014
CD1c	L161	Mouse								APC		Biolegend	331524
CD304	12C2	Mouse								BV421		Biolegend	354514
CD11c	3.9	Mouse								PerCP/Cy5.5		Biolegend	301624
CD86	IT2.2	Mouse								PE-Dazzle594		Biolegend	305434
CD56	NCAM16.2	Mouse							BUV395			BD	563554
