# Supplementary material for: Capturing the systemic immune signature of a norovirus infection: an n-of-1 case study within a clinical trial
Source: Wellcome Open Res. 2017 Oct 5;2:28. Originally published 2017 Apr 18. [Version 3] doi: 10.12688/wellcomeopenres.11300.3 (PMC5531165; doi:10.12688/wellcomeopenres.11300.3)
Supplement: Peer review history

# Peer review: Referee response for version 2

**DOI:** 10.21956/wellcomeopenres.13150.r24308

**Published:** 2017-07-26

**Authors:** Abimbola O. Kolawole

**Affiliations:** 1 Department of Microbiology and Immunology, University of Michigan Medical School, Ann Arbor, MI, USA

The authors have sufficiently improved the discussion section.

I have read this submission. I believe that I have an appropriate level of expertise to confirm that it is of an acceptable scientific standard.

# Peer review: Referee response for version 1

**DOI:** 10.21956/wellcomeopenres.12193.r22909

**Published:** 2017-06-13

**Authors:** Abimbola O. Kolawole

**Affiliations:** 1 Department of Microbiology and Immunology, University of Michigan Medical School, Ann Arbor, MI, USA

This manuscript presents a detailed characterization of the immune response to a possible human norovirus infection of a participant within a clinical trial.  Only one infected person and 5 non-infected controls were investigated. All the 6 tested participants were diabetic. No non-diabetic norovirus infected or non-infected person was included in the study. Despite all the limitations, the reported observations are relevant to the understanding of norovirus biology.

 Minor comments:

 1) The authors gave the impression that human norovirus infects only enterocytes. However, reports have shown that it also infects immune cells.

 2) The study will benefit from including the controls that were previously mentioned.

I have read this submission. I believe that I have an appropriate level of expertise to confirm that it is of an acceptable scientific standard, however I have significant reservations, as outlined above.

# Response

**Published:** 2017-07-11

**Authors:** Antony Cutler

**Affiliations:** University of Oxford, UK

*Ref 1:*

*This manuscript presents a detailed characterization of the immune response to a possible human norovirus infection of a participant within a clinical trial.  Only one infected person and 5 non-infected controls were investigated. All the 6 tested participants were diabetic. No non-diabetic norovirus infected or non-infected person was included in the study. Despite all the limitations, the reported observations are relevant to the understanding of norovirus biology.*

**

*Minor comments:*

*1) The authors gave the impression that human norovirus infects only enterocytes. However, reports have shown that it also infects immune cells.*

 We have adjusted the text accordingly and now reflect the observations that Norovirus capsid protein can be detected in immune cells.

 “Norovirus capsid protein can be detected in cells of the immune system such as T cells, macrophages and dendritic cells [ 29, 30] but appears to actively replicate in enterocytes *in vivo* and *in vitro* [ 29, 31]. “

*2) The study will benefit from including the controls that were previously mentioned.*

 We acknowledge that our study is limited to the study of individuals with type 1 diabetes within the clinical trial DILT1D. No contemporaneous non-diabetic controls were included in the study design, the primary aim of which was to determine the single dose of Proleukin required to increase Treg frequencies 10-20% above baseline levels. We agree that healthy controls with or without norovirus infection sampled longitudinally in the same intensive protocol would be a very valuable addition to the study and would allow comparison of responses between the two groups. We are limited though to the material from participants from the trial and can not add controls to the analysis.  

# Peer review: Referee response for version 1

**DOI:** 10.21956/wellcomeopenres.12193.r22905

**Published:** 2017-06-06

**Authors:** Jacob Kocher

**Affiliations:** 1 Department of Epidemiology, University of North Carolina at Chapel Hill, Chapel Hill, NC, USA

The present study features a thorough case study report on a human norovirus infection during a clinical trial. Given the obvious limitation on the number of NoV-infected patients, I feel the data falls into the "signal" end of the "signal:noise" ratio and is worthy to our understanding of immune responses to human NoV infection.

 However, I feel the manuscript could be improved with a few minor discussion points.

 1) The presented data varies between total concentration of cytokine and % shift above baseline, even within individual figures. Presumably, the cytokine responses were too low so total concentration would not be valuable for interpretation. The paper would benefit from consistent use of the % shift above baseline data.

 2) Diabetes contains numerous autoimmune characteristics, particularly in Treg cells. The manuscript would benefit from a discussion on how these autoimmune cell types may have contributed to the witnessed phenotypes. That is, did the combination of autoimmune disorder, infection, and IL-2 administration augmented the witnessed phenotypes?

I have read this submission. I believe that I have an appropriate level of expertise to confirm that it is of an acceptable scientific standard.

# Response

**Published:** 2017-07-11

**Authors:** Antony Cutler

**Affiliations:** University of Oxford, UK

*Ref 2:*

*The present study features a thorough case study report on a human norovirus infection during a clinical trial. Given the obvious limitation on the number of NoV-infected patients, I feel the data falls into the "signal" end of the "signal:noise" ratio and is worthy to our understanding of immune responses to human NoV infection.*

*However, I feel the manuscript could be improved with a few minor discussion points.*

*1) The presented data varies between total concentration of cytokine and % shift above baseline, even within individual figures. Presumably, the cytokine responses were too low so total concentration would not be valuable for interpretation. The paper would benefit from consistent use of the % shift above baseline data.*

Figure 2 does switch between total concentration of cytokine (panels A and B) and % change from baseline (panels C – I). We opted to use the % change from baseline in most cases as the levels of cytokine varied between the participants and for clarity we decided to standardize the starting levels to baseline. In general the concentrations of cytokines were above the levels of detection of the assays.  IL-12p70 is one example where they were not. Four out of the five controls had no detectable IL-12p70 at any point in the study, therefore we decided to present the data as total concentration rather than change from baseline *. *IL-10 levels were at the limit of detection of the MSD V-plex assay but we were confident  presenting them as % change from baseline as comparable concentrations of cytokine were detected when we tested the majority of samples in a high sensitivity S-plex MSD assay. Unfortunately we did not test the IL-10 levels of the norovirus infected individual as we had no remaining serum or plasma aliquots and therefore the data were not presented in this manuscript.

We presented the changes in IL-2 levels in concentration of cytokine again for reasons of clarity. Injection of Proleukin increases the levels of IL-2 in the plasma approaching 400,000 % above baseline. The secondary peak in IL-2 levels were 100,000 % above baseline in the norovirus infected individual. We can present the IL-2 data as a percentage change from baseline if required and have created an updated figure accordingly. Please Click here to access the figure. However we believe that the original presentation is most informative and would prefer to present the data in this way.  

*2) Diabetes contains numerous autoimmune characteristics, particularly in Treg cells. The manuscript would benefit from a discussion on how these autoimmune cell types may have contributed to the witnessed phenotypes. That is, did the combination of autoimmune disorder, infection, and IL-2 administration augmented the witnessed phenotypes?*

This is a very interesting question and one that is very hard to address without healthy individuals with or without norovirus infection and much larger sample sizes. We have added additional text to the discussion to highlight this interesting aspect of the work.  

“Despite the limitation of our study to individuals with type 1 diabetes participating in a clinical trial, detailed insights into the biology of Tregs in infection were achieved. Tregs are a key immune cell subset critical to maintaining immune tolerance, and have been considered to be defective in type 1 diabetes [37].  The increase in Treg frequency following norovirus infection was consistent with observations from studies in non-diabetic individuals with acute and chronic viral infections, malaria, and bacterial or fungal sepsis [38-44]. However in the absence of healthy controls and additional T1D patients with or without norovirus infection we cannot determine whether the regulatory or effector responses to infection are optimal in our diabetic participants. Modelling disease outcomes in mice has highlighted the complex inter-relationship between effector function and immune regulation by Tregs in response to infection. Rather than suppressing immune responses, Tregs were shown to coordinate immunity at the mucosal site of infection [45] and facilitate the generation of memory CD8 ^+^ T cells [46,47] in acute viral infections models.  The disease course was of normal duration in the affected participant and we propose that Proleukin administration was not detrimental to the response to norovirus in our trial participant”.

# Response

**Published:** 2017-07-11

**Authors:** Antony Cutler

**Affiliations:** University of Oxford, UK

The last sentence of the final edited paragraph was not correct. It should read "The disease course was of normal duration in the affected participant and, clinically, Proleukin administration did not affect the response to norovirus".
